# Digital health economics education: perspectives, potential and barriers at German medical universities

**DOI:** 10.3389/fmed.2025.1624347

**Published:** 2025-08-01

**Authors:** Stefan Hertling, Oliver Schöffski, Isabel Graul, Ekkehard Schleußner

**Affiliations:** ^1^Department of Obstetrics and Gynecology, Jena University Hospital, Jena, Germany; ^2^Department of Health Economics, University of Erlangen-Nuremberg, Nuremberg, Germany; ^3^Department of Trauma, Hand- and Reconstructive Surgery, University of Jena, Jena, Germany

**Keywords:** medical education, students, deans, medical universities, digital health economics education, health economics education, health economic

## Abstract

**Background:**

The increasing economization of healthcare systems highlights the need to integrate health economics more systematically into medical education.

**Objective:**

This study examines the perspectives of deans of German medical faculties on the integration of health economics content into medical studies. It also focuses on determining their ideas about suitable teaching formats and identifying potential obstacles to implementation. A particular and paradoxical finding of the study is the unanimous rejection of digital teaching formats by all participating faculties.

**Materials and methods:**

The pilot cross-sectional survey was conducted between April and October 2021. Deans and academic representatives from all 36 medical faculties in Germany were invited to participate. Data was collected using two specially developed standardized questionnaires with 24 questions.

**Results:**

A total of 28 medical faculties participated in the survey. 88% of respondents do not currently teach health economics, although 92% consider teaching health economics in medical studies to be important. The main obstacles cited are the lack of recognition as a medical discipline (96%), the absence of a conceptual curriculum (77%) and a lack of teaching capacity and financial resources (77 and 62% respectively). 81% see a conceptual curriculum as a prerequisite for national standardization. 38% call for interdisciplinary cooperation with health economics faculties.

## Introduction

The economic conditions in the healthcare sector are undergoing rapid change (1). Rising costs, increasing pressure on resources, and structural deficits in many institutions also require physicians to have a growing understanding of health economics (2). Although medical decisions in all areas of healthcare are increasingly influenced by economic factors—such as the DRG system, drug budgets, and staff allocation—this reality is hardly reflected in the curriculum for prospective doctors (3).

In view of the increasing economic challenges and structural changes in the healthcare system, teaching health economics skills in medical studies is becoming increasingly important. It is important to distinguish between basic health economics education, which imparts knowledge of health economics, and digital health economics education. The latter uses digital teaching formats such as e-learning, online seminars, or blended learning to convey this content. This conceptual diversification is necessary because content and didactic implementation take place at divergent levels and each present specific challenges and potential (4, 5).

At the international level, there is a trend toward a growing number of countries—including the United States, Canada, the United Kingdom, and Scandinavian countries—integrating health economics content into their medical curricula in recent years. In the United Kingdom, for example, the subject of health economics is an integral part of the public health-oriented training segment. In the United States, too, numerous medical schools offer electives or specializations that shed light on economic decision-making processes in the healthcare system. In Scandinavian countries, on the other hand, the interdisciplinary approach between medicine, economics, and healthcare research is particularly strong (6, 7). The international examples available underscore the relevance of the topic and provide valuable benchmarks for assessing the situation in Germany (8). In fact, there is no nationwide curriculum in Germany that structurally provides for the teaching of health economics in medical studies (9). The content is determined by the individual faculties. Health economics topics—if they are covered at all—are usually taught in voluntary additional courses (10). This internal faculty structure results in a heterogeneous educational situation and prevents the systematic development of skills in an area that is becoming increasingly important for the medical profession (11).

In view of the significant challenges currently facing medical faculties, such as the economic difficulties of many hospitals, demographic change, and advancing digitalization, the question arises as to how health economics education should be integrated into medical studies. This concerns aspects such as structure, teaching methods, and digitalization (12). The discussion about potential curricular integration is accompanied by structural obstacles such as limited teaching capacities, a lack of conceptual curricula, and insufficient specialist resources (13, 14).

This study examines the assessments and experiences of deans of German medical faculties regarding the role of digital health economics education as a forward-looking teaching concept in medical studies. Another focus is on the obstacles that stand in the way of its implementation in the curriculum.

## Materials and methods

### Ethics commission

The responsible Ethics Committee of the University of Jena was informed and did not raise any objections to the study (Reg. -Nr.: 2019-1456-Bef).

### Study setting

This pilot study was conducted as part of an interdisciplinary collaboration between the medical faculty of Jena University Hospital and the Chair of Health Economics at FAU Erlangen. The survey was distributed to all 36 medical faculties in Germany (15). The empirical survey was conducted between April and October 2021. The questionnaires were sent to the deans of the medical faculties. This was done via publicly available email addresses of the vice deans or their secretariats. Potential participants were informed about the content and purpose of the survey in an information letter attached to the email. The document also stated that the data would be treated as strictly confidential and anonymized. Participants were given access to the study via a survey link in the cover letter. Before starting the survey, participants had to actively confirm their participation and consent to the anonymous storage of their data.

### Sample sizes

Twenty-eight responses were received from the deans, corresponding to a response rate of just under 78%.

### Design of the questionnaires

The questionnaire was developed in close cooperation with the Chair of Health Economics at Friedrich-Alexander University Erlangen specifically for the research questions of the study. The questionnaire was tested in several steps and adapted in pilot interviews within the research team to ensure its quality and comprehensibility. The drafts were based on published guidelines for questionnaire research (16) and were created in a web-based design, with technical implementation carried out using SurveyMonkey (SurveyMonkey, San Mateo, CA). The questions for the study were selected based on a careful analysis of comparable scientific studies and additionally underpinned by quality criteria for online questionnaires (17, 18, 19). To ensure the highest possible validity and comprehensibility of the questions, the pilot questionnaires were tested on 20 randomly selected medical students. The feedback received was considered and used to further optimize the survey elements.

Each of the two questionnaires consisted of 24 questions divided into four subject areas. The possible answers to the questions were based on a five-point Likert scale, which offered respondents the following options: ‘strongly agree’ (1), ‘agree’ (2), ‘neutral’ (3), ‘disagree’ (4) and ‘strongly disagree’ (5). In addition, the questionnaires contained open-ended questions that allowed participants to provide comments and further remarks.

The four main topics of the questionnaires were as follows:

Information about the respondents.
Attitudes toward health economics.
Questions about the introduction of health economics into the human medicine curriculum.
Barriers and attitudes toward the introduction of health economics as a subject of study.

The questionnaire was entitled ‘Health economics in human medicine’. The primary goal of the surveys was to keep the response time as short as possible (approx. 15 min) to ensure a high participation rate and a low dropout rate, while at the same time enabling thorough and detailed answers to the questions (20).

Handling of missing data: Individual incomplete questionnaires or unanswered items were excluded from the respective analysis (“listwise deletion”), but documented in the overall evaluation. The survey instruments were designed to enable systematic and uniform evaluation using closed response formats.

Limitations of the methodology: As this is a cross-sectional study, the data collected can only reflect the views of the respondents at the time of the survey. Due to the design, it is not possible to draw causal interpretations or infer developments. Furthermore, selection bias cannot be completely ruled out, as not all faculties responded.

### Inclusion and exclusion criteria

Participants had to be at least 18 years old and work as a dean or vice-dean of a medical faculty in Germany. To ensure data quality, only completely and correctly completed questionnaires were included in the study.

Of the 36 medical faculties, a total of 28 participated. The response rate was therefore 78% (28/36). Of the 28 completed questionnaires, two had to be excluded as incomplete. The response rate was therefore 72% (26/36).

### Data analysis

Only fully completed questionnaires were included in the subsequent data analysis. The responses were evaluated using the SurveyMonkey platform and the statistical program SPSS (version 17.0, SPSS Inc., Chicago, IL, United States). The evaluation was carried out using non-parametric statistical tests. Due to the predominantly ordinal scale data and the limited number of cases, parametric test procedures were not used. Instead, the Mann–Whitney U test was used to analyze two independent groups, the chi-square test was used to examine frequency distributions, and the binomial test was used to test against hypothetical distributions. These tests were chosen because of the distribution characteristics of the data collected and the objective of the study, which was to enable robust statements to be made without assuming a normal distribution.

## Results

### Epidemiological data from medical deans

22 of the 26 respondents (85%) provided information about their current position at the medical faculty. The teaching staff was composed as follows: 10/22 were doctors in patient care (45%), 3/22 were doctors with exclusively administrative tasks (14%) and 9/22 were university employees without a medical license (41%). 12/22 of the respondents had dealt with health economic issues very frequently or frequently during their professional career (55%). Ten out of 22 respondents stated that they had rarely been confronted with health economics issues in their professional career to date (45%). 287 out of 22 respondents stated that they had completed additional training in health economics (31%). Of the seven respondents, four had completed additional studies (4/22, 18%), two had a university degree in economics/business administration (2/22, 9%) and one respondent stated that they had completed further training in health management (1/22, 4.5%) (see Table 1).

**Table 1:** Faculty respondent characteristics and statistical significance.

Category	Number	Percent	*p*-value (vs. 50%)
Physicians in patient care	10	45.5%	0.83181
Physicians with administrative duties	3	13.6%	0.00086
Non-physician staff	9	40.9%	0.52347
Frequently faced health economics questions	12	54.5%	0.83181
Rarely faced health economics questions	10	45.5%	0.83181
Any qualification in health economics	7	31.8%	0.1338
Postgraduate studies in health economics	4	18.2%	0.00434
Degree in business/economics	2	9.1%	0.00012
Further training in health management	1	4.5%	0.00001

### Status quo of health economics teaching at medical faculties

**Figure 1:**
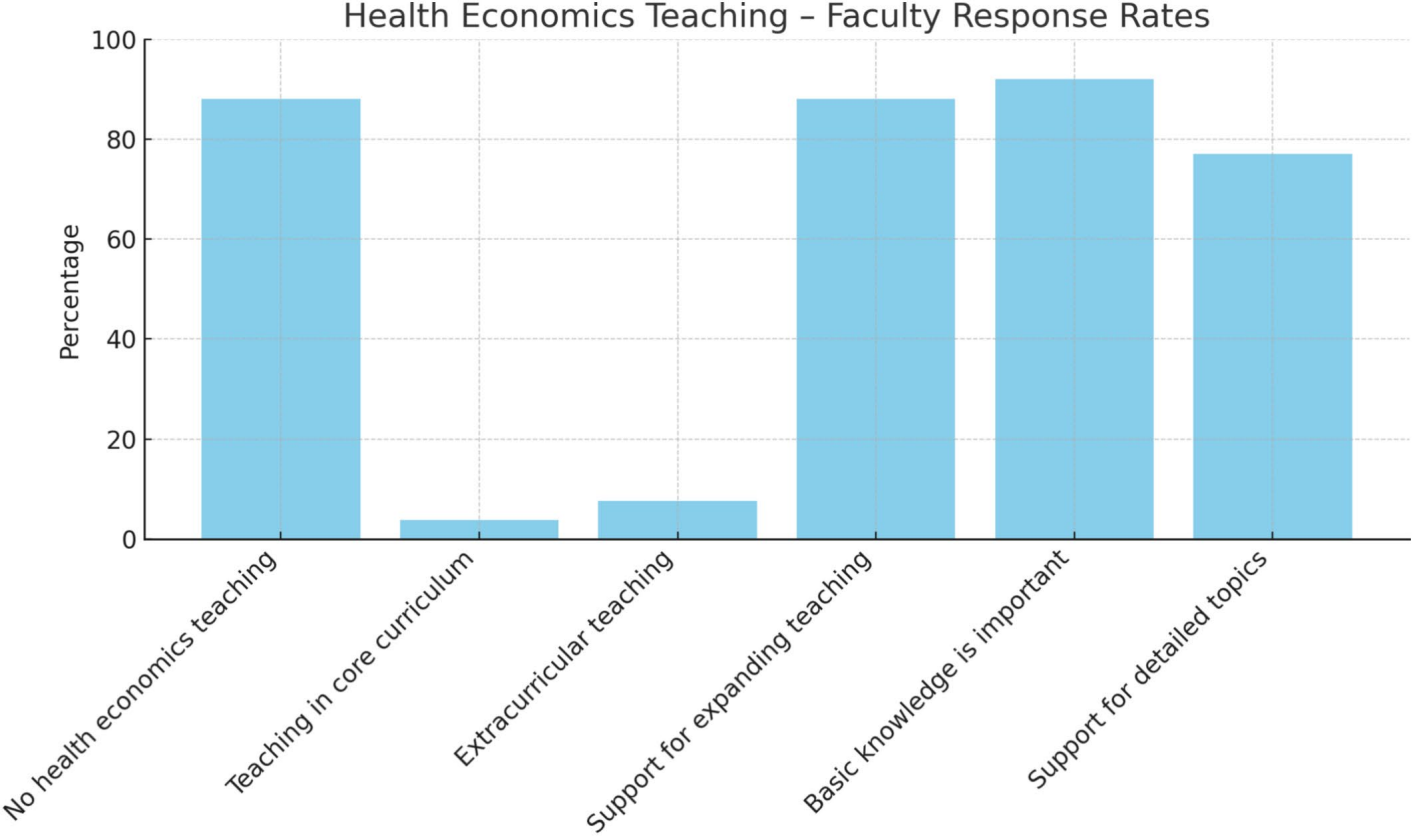
Health economics teaching – faculty response rates.

Respondents indicated whether health economics teaching formats are already part of human medicine teaching at their medical school. The data show that 23 of the 26 participating medical schools (88%) do not offer health economics teaching. Three of the 26 participating medical schools offer teaching formats with a focus on health economics: one medical faculty offers a compulsory elective seminar in the clinical study section (health economics, health management, public health) as part of the core curriculum (3.8%), and two of the 26 medical faculties teach health economics in extracurricular courses (7.7%). At the two medical faculties, teaching takes the form of additional elective courses that students can take voluntarily. Overall, lectures and online seminars are the most frequently used teaching formats at the three medical faculties where health economics is taught in medical education. Participants were then asked whether they would support an expansion of health economics teaching. Twenty-three of the 26 medical faculties (88%) were in favor of this. Twenty-four of the 26 (92%) agreed that learning the basics of health economics is important for every medical student. The teaching of detailed aspects of health economics was also supported by a large majority of medical faculties (20/26, 77%) (see Figure 1). Most medical faculties consider the teaching of health economics in the clinical section to be appropriate (19/26, 73%). Three of the medical faculties would start teaching earlier (3/26, 11.5%). Regarding the duration of teaching health economics, 20 of 26 medical faculties (77%) stated that the duration is one semester (see Table 2).

**Table 2:** Status quo of health economics teaching at medical faculties.

Category	Percentage	Binomial *p*-value (vs. 50%)	Significance
No health economics teaching	88%	0.00009	Significant
Teaching in core curriculum	3.8%	1.0	Not significant
Extracurricular teaching	7.7%	1.0	Not significant
Support for expanding teaching	88%	0.00009	Significant
Basic knowledge is important	92%	0.00001	Significant
Support for detailed topics	77%	0.00446	Significant

Since health economics is not a separate subject in Germany, medical faculties were asked to name the subject areas that should be primarily responsible for health economics education: general medicine (12/26, 46%), occupational and environmental medicine (7/26, 27%) and geriatrics (4/26, 15%). When asked whether cooperation with health economics organizations would need to be established to establish teaching content, only two of the 26 medical faculties (7.7%) responded that they would rule out such cooperation. Three of the medical faculties surveyed also stated in the comments section of the questionnaire that their faculty already had cooperation agreements with health economics institutions (3/26, 12%), without specifying the exact content.

Expansion of teaching in the field of health economics in human medicine studies.

A total of 23 of the 26 medical faculties (88%) agreed to include health economics in the medical faculty curriculum. In the present study, 21 of the 26 medical faculties (80%) identified the tension between the already increasing workload of medical students and the urgency of implementing health economics as the greatest challenge.

**Figure 2:**
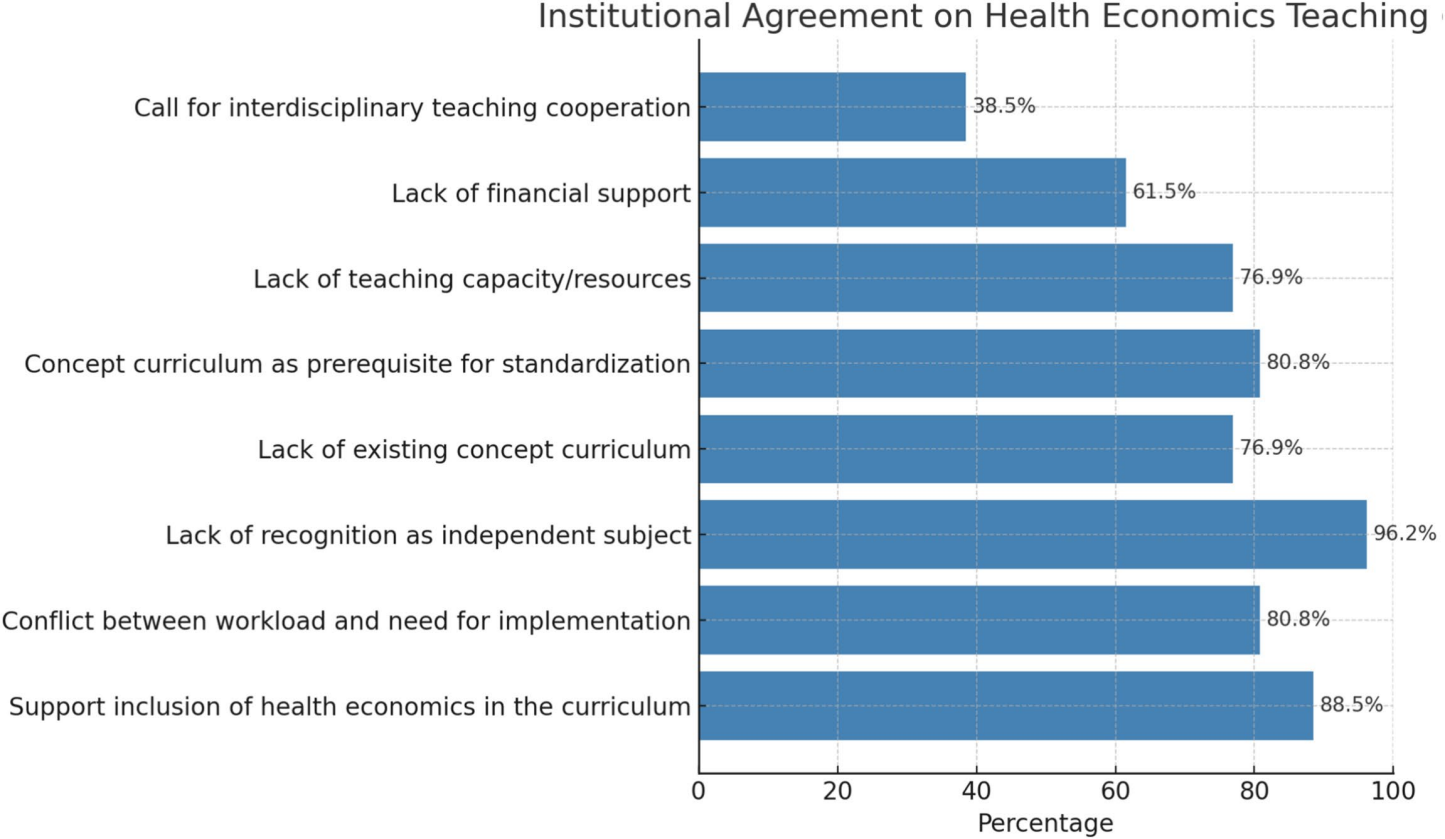
Institutional support and barriers for health economics teaching.

Most medical faculties (25/26, 96%) cited the lack of recognition as an independent medical discipline as a further obstacle to its introduction. Furthermore, there is a lack of an existing concept curriculum that could serve as a basis for the curricular design. This deficiency was highlighted by 77% of respondents. In the present study, 21 of the 26 medical faculties surveyed (81%) agreed that the curriculum concept is a prerequisite for nationwide standardization. Other obstacles to the expansion of teaching in the field of health economics include a lack of teaching capacity and resources (20/26, 77%) and a lack of financial support (16/26, 62%). In addition, 10 of the 26 medical faculties called for interdisciplinary teaching cooperation with departments focusing on health economics (10/26, 38%) (see Table 3; Figure 2).

**Table 3:** Significance of institutional responses to expanding health economics teaching.

Category	Percentage	*p*-value (Binomial test vs. 50%)	Significant?
Support inclusion of health economics in the curriculum	88.5%	9e-05	Yes
Conflict between workload and need for implementation	80.8%	0.00249	Yes
Lack of recognition as independent subject	96.2%	0.0	Yes
Lack of existing concept curriculum	76.9%	0.00936	Yes
Concept curriculum as prerequisite for standardization	80.8%	0.00249	Yes
Lack of teaching capacity/resources	76.9%	0.00936	Yes
Lack of financial support	61.5%	0.32694	No
Call for interdisciplinary teaching cooperation	38.5%	0.32694	No

### Options for teaching health economics

**Figure 3:**
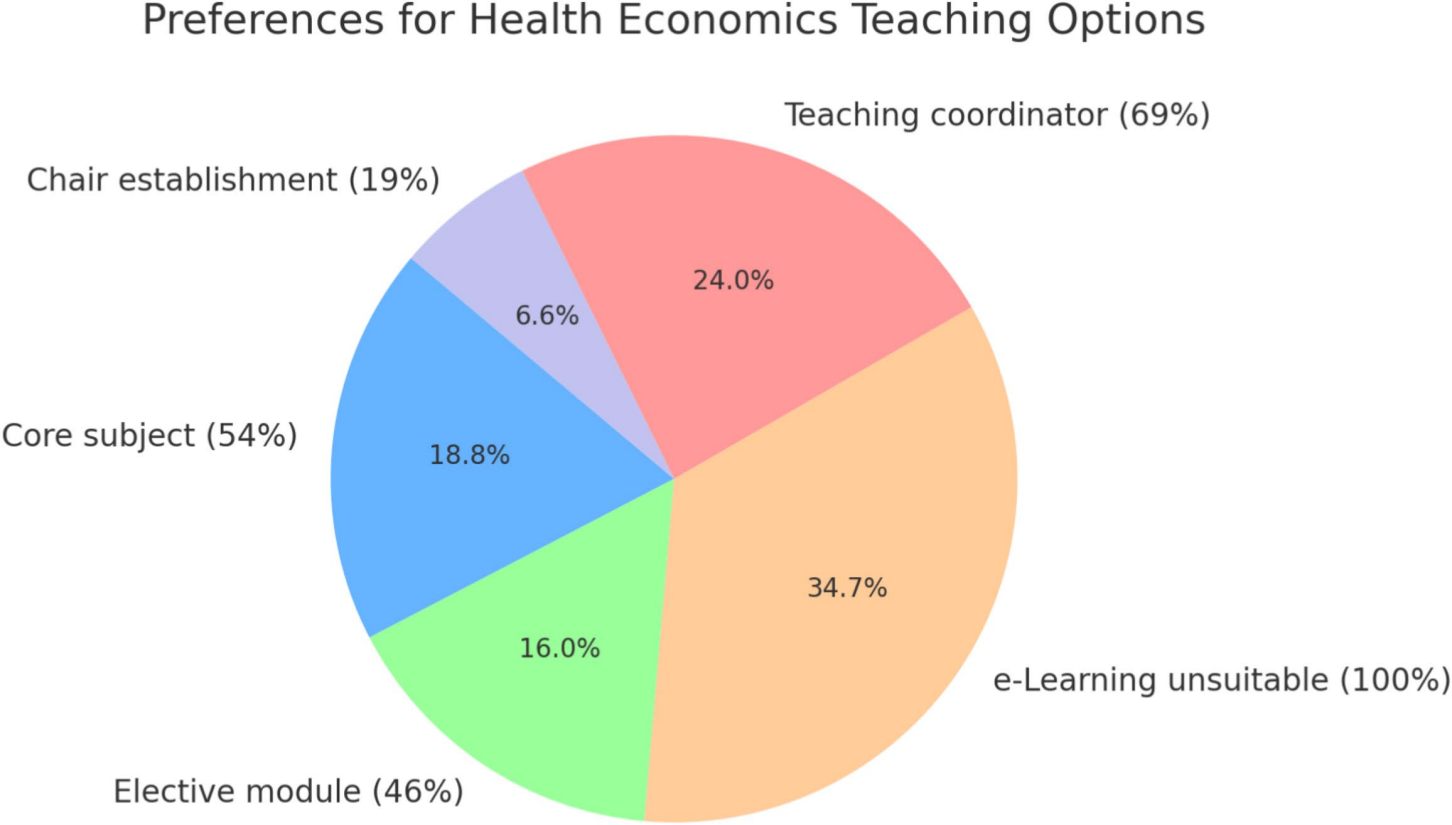
Preferences for health economics teaching options.

It is the responsibility of medical faculties to evaluate teaching methods that are suitable for implementing health economics in their curricula. The present study found that 54% of the 26 medical faculties were in favor of implementing health economics as a separate subject in the regular curriculum. 12 of the 26 medical faculties support the introduction of health economics as a compulsory elective module, which corresponds to a rate of 46%. Furthermore, all medical faculties rated e-learning as unsuitable for health economics, corresponding to a rate of 100%. There is no evidence that a chair for health economics has been established at any medical faculty in Germany. However, one faculty reported that an institute for health economics had been established. It can be concluded that 18 of the 24 medical faculties (69%) were in favor of appointing a teaching supervisor, while five faculties (19%) saw a need to establish a chair in health economics (see Table 4; Figure 3).

**Table 4:** Preferences and significance – teaching options in health economics.

Category	Percentage	Binomial *p*-value (vs. 50%)	Significant?
Support integration as core subject	53.8%	0.84502	No
Support integration as elective module	46.2%	0.84502	No
e-Learning rated unsuitable	100.0%	0.0	Yes
Support appointment of teaching coordinator	75.0%	0.02266	Yes
Support establishment of a chair	19.2%	0.00249	Yes

### Evidence of divergences in the evaluation of teaching formats in health economics

The pairwise chi-square comparisons of the various teaching options in the field of health economics reveal clear differences in the evaluation by medical faculties. Particularly striking is the consistently significant deviation in the assessment of e-learning compared to all other teaching options. The unanimous rejection of digital formats (100% agreement on unsuitability) differs statistically significantly from all other variants – an indication of institutionally anchored skepticism toward digital teaching in this subject area.

The comparison between integration as a compulsory subject (core subject) and the establishment of a chair is also significant. While a narrow majority is in favor of integration into the curriculum (54%), only 19% of faculties see the need to establish a separate chair. This points to a contradiction between content relevance and structural anchoring.

The remaining comparisons – for example, between elective modules and compulsory subjects or between coordinated teaching and the establishment of chairs – were not statistically significant. This shows that different forms of embedding health economics content are largely considered equivalent by the faculties if they are not digital (see Table 5).

**Table 5:** Mismatch analysis: teaching option comparisons (Chi^2^).

Comparison	*p*-value (Chi^2^)	Significant
Core subject vs. Elective module	0.78151	No
Core subject vs. e-Learning unsuitable	0.00029	Yes
Core subject vs. Teaching coordinator	0.20694	No
Core subject vs. Chair establishment	0.02123	Yes
Elective module vs. e-Learning unsuitable	5e-05	Yes
Elective module vs. Teaching coordinator	0.07326	No
Elective module vs. Chair establishment	0.0761	No
e-Learning unsuitable vs. Teaching coordinator	0.02247	Yes
e-Learning unsuitable vs. Chair establishment	0.0	Yes
Teaching coordinator vs. Chair establishment	0.00024	Yes

## Discussion

This study provides comprehensive insights into the current situation and assessments of medical faculties in Germany regarding health economics education in human medicine. The focus is on the concept of ‘digital health economics education,’ which describes the digital, systematic, and interdisciplinary teaching of health economics skills as part of medical training. In view of the increasing economic challenges facing the healthcare system, this topic is becoming increasingly relevant – both for healthcare practice and for the curriculum design of medical degree programs (21, 22, 23).

The study results show a high level of fundamental agreement on the relevance of health economics content: 88% of the faculties surveyed are in favor of incorporating it into the curriculum, and 92% consider teaching the fundamentals of health economics to be essential. At the same time, 80% identify a tension between the increased workload of students and the need to expand the curriculum. This assessment is in line with current findings on the challenges in medical studies, which are characterized by the densification of the curriculum and the associated psychological strain on students (24, 25).

The lack of recognition of health economics as an independent medical discipline was identified as a significant structural barrier (96%). In addition, there is a lack of a conceptual curriculum that could serve as a foundation for standardizing content. The demand for such a concept to support nationwide implementation met with broad approval (81%). Similar challenges have been described in other countries, where health economics content was initially integrated through pilot programs and only later established in the curriculum (26, 27).

Of interest is the significant rejection of digital teaching formats: the faculties surveyed rated e-learning as unsuitable. This attitude contradicts the broad acceptance of digital education formats in higher education in general and reveals a discrepancy between technical possibilities and professional acceptance (28). The development of educational programs in the field of digital health economics education could help to efficiently address existing deficits in teaching without further condensing the structure of the curricula through the implementation of flexible, scalable and interactive formats (29).

The analysis of significant differences between different teaching models also reveals a clear mismatch: while more than half support integration as a compulsory subject or elective module, only 19% are in favor of establishing a chair. This structural reluctance suggests that health economics content is considered relevant but is not (yet) to be institutionally anchored. This hinders sustainable integration and contradicts the requirement for a long-term education strategy (30).

To establish digital health economics education as a fixed component of medical training, a nationally coordinated concept curriculum is therefore required, as well as targeted structural measures: interdisciplinary cooperation with economics departments, start-up funding for innovative teaching formats and incentives for teaching staff to obtain further qualifications. Such a change must meet both the content requirements and the reality of medical education to have a lasting effect (31).

The results of this study underscore that there is no uniform consensus within the faculty landscape on the optimal teaching format, but there is clear rejection of e-learning and structural reluctance regarding permanent institutional anchoring such as chairs.

The results of this study not only highlight the structural and content-related challenges involved in integrating health economics into medical curricula but also reveal a fundamental tension between the need for innovation and institutional inertia. Of particular interest is the unanimous rejection of digital teaching formats by the medical faculties surveyed. This attitude appears inconsistent in the context of digitization strategies in the education sector and points to cultural barriers within medical universities (32).

One possible explanation for the reluctance to embrace digital teaching methods could be the low level of digital teaching skills among many teachers. According to several international studies, the use of digital tools in medical education is rejected, especially in cases where there is a lack of methodological support and subject-specific training (33, 34). A certain methodological conservatism, which is particularly prevalent in traditional medical schools, is often accompanied by a preference for face-to-face teaching and a narrow understanding of scientific rigor.

Considering this, it seems sensible to discuss concrete reform approaches:

Interdisciplinary structures can be promoted through systematic cooperation between medical and economics faculties. Such cooperation could strengthen health economics expertise and enable new teaching formats. Possible options include joint teaching assignments, shared professorships, or modular curricula (35).

The acquisition of structural funds and third-party funding is an essential aspect of research funding. The financing of educational innovations in the field of digital health economics education (DHEE) could be targeted through funding programs at the national or international level (36).

The implementation of digital health economics content in the accreditation requirements of medical faculties could potentially lead to standardization. A stronger link to the revised medical licensing regulations could provide an additional incentive for integration (37).

The implementation of these reform options requires a rethinking of university culture. The increasing digitalization and economization of the healthcare system is leading to a high demand for health economics skills. In view of this, it seems urgent to remove structural and cultural barriers and prepare academic medicine for the future (38).

## Conclusion

This study concludes that a significant number of medical schools are aware of the relevance of health economics content in medical education and support its integration into the curriculum. At the same time, there are structural barriers, such as the lack of recognized professional accreditation, insufficient teaching resources, and the absence of a conceptual curriculum.

What is striking is the unanimous rejection of digital teaching formats by the faculties surveyed, which contrasts with the general acceptance of digital teaching in higher education. However, this challenge also presents an opportunity: digital health economics education can make a lasting contribution to the modernization of medical education. Digital formats enable flexible, resource-efficient, and scalable teaching of health economics content, thus offering a solution to the overloaded curricula of many degree programs.

Greater openness on the part of medical schools to interdisciplinary and digital teaching concepts could not only contribute to improving the teaching of health economics but also ensure that it becomes firmly anchored in medical education. The future of health economics teaching lies in a combination of professional relevance, digital innovation, and institutional openness.

### Limitations of the study and outlook for further research

The present study has several limitations. The sample size is limited and refers exclusively to German medical schools, which restricts the transferability of the results to international contexts. Furthermore, it should be noted that this is a cross-sectional design that only provides a snapshot of attitudes without establishing causal links between attitudes, institutional frameworks, and curricular implementation.

The following aspects should play a central role in future research:

Longitudinal studies will measure the effects of health economics teaching on the skills, career paths, and decision-making behavior of medical students.
International comparative studies have examined the integration of health economics into the curricula of medical degree programs.

This study will conduct evaluation studies on the effectiveness of different teaching formats. To this end, digital versus analog and interdisciplinary versus monodisciplinary teaching formats will be compared. This study analyzes cultural and structural barriers that play a significant role in the implementation of digital educational content in the medical sector. Such a research focus can contribute to the development of evidence-based strategies for the sustainable implementation of health economics education in medicine.

## Data Availability

The original contributions presented in the study are included in the article/supplementary material, further inquiries can be directed to the corresponding author/s.
